# Acute Human Lethal Toxicity of Agricultural Pesticides: A Prospective Cohort Study

**DOI:** 10.1371/journal.pmed.1000357

**Published:** 2010-10-26

**Authors:** Andrew H. Dawson, Michael Eddleston, Lalith Senarathna, Fahim Mohamed, Indika Gawarammana, Steven J. Bowe, Gamini Manuweera, Nicholas A. Buckley

**Affiliations:** 1 South Asian Clinical Toxicology Research Collaboration, Faculty of Medicine, University of Peradeniya, Peradeniya, Sri Lanka; 2 School of Medicine and Public Health, University of Newcastle, Newcastle, Australia; 3 Professorial Medicine Unit, POW Clinical School, University of New South Wales, Sydney, Australia; 4 National Poisons Information Service - Edinburgh, Royal Infirmary of Edinburgh, Edinburgh, United Kingdom; 5 Clinical Pharmacology Unit, Centre for Cardiovascular Science, University of Edinburgh, Edinburgh, United Kingdom; 6 Office of the Pesticide Registrar, Government Department of Agriculture, Peradeniya, Sri Lanka; University College London, United Kingdom

## Abstract

**Background:**

Agricultural pesticide poisoning is a major public health problem in the developing world, killing at least 250,000–370,000 people each year. Targeted pesticide restrictions in Sri Lanka over the last 20 years have reduced pesticide deaths by 50% without decreasing agricultural output. However, regulatory decisions have thus far not been based on the human toxicity of formulated agricultural pesticides but on the surrogate of rat toxicity using pure unformulated pesticides. We aimed to determine the relative human toxicity of formulated agricultural pesticides to improve the effectiveness of regulatory policy.

**Methods and Findings:**

We examined the case fatality of different agricultural pesticides in a prospective cohort of patients presenting with pesticide self-poisoning to two clinical trial centers from April 2002 to November 2008. Identification of the pesticide ingested was based on history or positive identification of the container. A single pesticide was ingested by 9,302 patients. A specific pesticide was identified in 7,461 patients; 1,841 ingested an unknown pesticide. In a subset of 808 patients, the history of ingestion was confirmed by laboratory analysis in 95% of patients. There was a large variation in case fatality between pesticides—from 0% to 42%. This marked variation in lethality was observed for compounds within the same chemical and/or WHO toxicity classification of pesticides and for those used for similar agricultural indications.

**Conclusion:**

The human data provided toxicity rankings for some pesticides that contrasted strongly with the WHO toxicity classification based on rat toxicity. Basing regulation on human toxicity will make pesticide poisoning less hazardous, preventing hundreds of thousands of deaths globally without compromising agricultural needs. Ongoing monitoring of patterns of use and clinical toxicity for new pesticides is needed to identify highly toxic pesticides in a timely manner.

*Please see later in the article for the Editors' Summary*

## Abstract 2

In a prospective cohort study of patients presenting with pesticide self-poisoning, Andrew Dawson and colleagues investigate the relative human toxicity of agricultural pesticides and contrast it with WHO toxicity classifications, which are based on toxicity in rats.

## Editors' Summary

**Background:**

Suicide is a preventable global public health problem. About 1 million people die each year from suicide and many more harm themselves but survive. Although many people who commit suicide have a mental illness, stressful events (economic hardship or relationship difficulties, for example) can sometimes make life seem too painful to bear. Suicide attempts are frequently impulsive and use methods that are conveniently accessible. Strategies to reduce suicide rates include better treatment of mental illness and programs that help people at high risk of suicide deal with stress. Suicide rates can also be reduced by limiting access to common suicide methods. The single most important means of suicide worldwide is agricultural pesticide poisoning. Every year, between 250,000 and 370,000 people die from deliberate ingestion of pesticides (chemicals that kill animal pests or unwanted plants). Most of these suicides occur in rural areas of the developing world where high levels of pesticide use in agriculture combined with pesticide storage at home facilitate this particular method of suicide.

**Why Was This Study Done?:**

To help reduce suicides through the ingestion of agricultural pesticides, the Food and Agriculture Organization of the United Nations recommends the withdrawal of the most toxic pesticides—World Health Organization (WHO) class I pesticides—from agricultural use. This strategy has proven successful in Sri Lanka where a ban on class I pesticides in 1995 and on the class II pesticide endosulfan in 1998 has reduced pesticide deaths by 50% over the past 20 years without decreasing agricultural output. Further reductions in suicides from pesticide ingestion could be achieved if regulatory restrictions on the sale and distribution of the most toxic class II pesticides were imposed. But such restrictions must balance agricultural needs against the impact of pesticides on public health. Unfortunately, the current WHO pesticide classification is based on toxicity in rats. Because rats handle pesticides differently from people, there is no guarantee that a pesticide with low toxicity in rodents is safe in people. Here, the researchers try to determine the relative human toxicity of agricultural pesticides in a prospective cohort study (a study in which people who share a characteristic—in this case, deliberate pesticide ingestion—are enrolled and followed to see how they fare).

**What Did the Researchers Do and Find?:**

The researchers examined the case fatality (the proportion of patients dying after hospital admission) of different agricultural pesticides among patients who presented with pesticide self-poisoning at two Sri Lankan referral hospitals. Between April 2002 and November 2008, 9,302 people were admitted to the hospitals after swallowing a single pesticide. The researchers identified the pesticide ingested in 7,461 cases by asking the patient what he/she had taken or by identifying the container brought in by the patient or relatives. 10% of the patients died but there was a large variation in case fatality between pesticides. The herbicide paraquat was the most lethal pesticide, killing 42% of patients; several other pesticides killed no one. Compounds in the same chemical class and/or the same WHO toxicity class sometimes had very different toxicities. For example, dimethoate and malathione, both class II organophosphate insecticides, had case fatalities of 20.6% and 1.9%, respectively. Similarly, pesticides used for similar agricultural purposes sometimes had very different case fatalities.

**What Do These Findings Mean?:**

These findings provide a toxicity ranking for pesticides that deviates markedly from the WHO toxicity classification based on rat toxicity. Although the findings are based on a study undertaken at just two Sri Lankan hospitals, they are likely to be generalizable to other hospitals and to other parts of rural Asia. However, because the study only included patients who were admitted to hospital after ingesting pesticides, the actual case fatalities for some pesticides may be somewhat different. Nevertheless, these findings have several important public health implications. For example, they suggest that the decision taken in January 2008 to withdraw paraquat, dimethoate, and fenthion from the Sri Lankan market should reduce deaths from pesticide poisoning in Sri Lanka by a further 33%–65% (equivalent to about 1,000 fewer suicides per year). More generally, they suggest that basing the regulation of pesticides on human toxicity has the potential to prevent hundreds and thousands of intentional and accidental deaths globally without compromising agricultural needs.

**Additional Information:**

Please access these Web sites via the online version of this summary at http://dx.doi.org/10.1371/journal.pmed.1000357.

- This study is further discussed in a *PLoS Medicine* Perspective by Matt Miller and Kavi Bhalla
- The World Health Organization provides information on the global burden of suicide and on suicide prevention (in several languages) and on its classification of pesticides
- The US Environmental Protection Agency provides information about all aspects of pesticides (in English and Spanish)
- Toxtown, an interactive site from the US National Library of Science, provides information on environmental health concerns including exposure to pesticides (in English and Spanish)
- The nonprofit organization Pesticide Action Network UK provides information about all aspects of pesticides
- The US National Pesticide Information Center provides objective, science-based information about pesticides (in several languages)
- The Food and Agriculture Organization of the United Nations leads international efforts to reduce hunger; as part of this effort, it has introduced pesticide policy reforms (in several languages)
- MedlinePlus provides links to further resources about suicide and about pesticides (in English and Spanish)

## Introduction

Suicide and deliberate self-harm using pesticides is a major but under-recognised public health problem in the developing world. Each year 250,000–370,000 thousand people die from deliberate ingestion of pesticides [1],[2]. These deaths are responsible for about a third of suicides globally [1]; the World Health Organization (WHO) now recognizes pesticide poisoning to be the single most important means of suicide worldwide [3].

Within the rural developing world, high levels of pesticide use with storage at home increases the risk of acute poisoning [4]. One strategy to reduce mortality is to restrict access to more toxic pesticides [5]. As a first line, countries should follow Food and Agriculture Organization (FAO) advice [6] and withdraw the most toxic WHO/US Environmental Protection Agency (EPA) class I pesticides (see Table 1 for description of toxicity classifications) from agricultural practice. Further efforts can range from educating farmers in the use of safer pesticides to imposing regulatory restrictions on the sale and distribution of the most toxic class II pesticides [7],[8]. These strategies would be aided if countries developed pesticide policies that balanced agricultural and economic needs against the public health impact of acute and chronic human toxicity [9]. The development of such a policy should be iterative and based on evidence about which pesticides are major public health concerns [1].

**Table 1:** WHO classification of toxicity [18].

Class	Description	LD_50_ for the Rat (mg/kg Body Weight)			
		Oral		Dermal	
		Solids	Liquids	Solids	Liquids
Ia	Extremely hazardous	≤5	≤20	≤10	≤40
Ib	Highly hazardous	5–50	20–200	10–100	40–400
II	Moderately hazardous	50–500	200–2,000	100–1,000	400–4,000
III	Slightly hazardous	>500	>2,000	>1,000	>4,000

Unfortunately, at present, regulatory decisions are based on a classification of pesticide toxicity that is largely based upon rat oral LD50s. The scientific basis for extrapolating this classification to human poisoning with class II pesticides is weak. Rodents handle xenobiotics differently to humans [10]; as an example, they have greater capacity for metabolic detoxification of organophosphates [11]. And unlike human patients receiving intensive care, they do not receive any treatment in the toxicity studies. It is therefore not clear that a pesticide with low toxicity in rodents should be safe in humans and vice versa.

Information on the acute human toxicity of a wide range of pesticides is needed to complement the animal toxicity data. We therefore set up a prospective cohort of intentional pesticide-poisoned patients admitted to two Sri Lankan hospitals to study the relative toxicity of formulated pesticides in humans. Sri Lanka is an ideal location for this research. Ahead of the FAO recommendations, it has banned all WHO/EPA class I pesticides leaving just class II and III pesticides in agricultural use. Other Asian countries are slowly following suit—within a few years class I pesticides will no longer be used. Therefore, the current situation in Sri Lanka represents the future situation in other Asia countries. Furthermore, because only class II and III pesticides are used in Sri Lanka, the great majority of deaths from pesticide poisoning occur in the secondary referral study hospitals [12],[13]. Bias from prehospital deaths is minimized.

## Methods

### Ethics Statement

Ethics approval for this data collection has been obtained from Oxfordshire Clinical Research Ethics Committee, Oxford Tropical Medicine Ethics Committee, Colombo University Faculty of Medicine Ethics Committee, Sri Lankan Medical Association, University of Peradeniya, and the Australian National University.

### Patients

This was a prospective observational cohort study of patients aged greater than 12 y with deliberate ingestion of a single pesticide who presented to two Sri Lankan rural referral hospitals for an agricultural provincial district of 1.1 million people. The poison ingested was determined from the history given by the patient or relatives, the hospital transfer letter, or from the pesticide bottle if it accompanied the patient. Patients who deliberately ingested more than one substance (except for alcohol) were excluded from this study. Patients were either direct admissions to the study hospital or transfers from smaller primary hospitals. Patient recruitment commenced on 31 March 2002 in Anuradhapura Hospital and 4 June 2002 in Polonnaruwa Hospital. Details of the referring hospital name were documented from 1 June 2006. Data analysis was performed on all patients who had been enrolled up until 17 November 2008.

All patients were enrolled into the cohort at admission by full-time study doctors employed as clinical research assistants. Clinical care followed a standard protocol that emphasized identifying patients poisoned by pesticides that would likely respond to antidotes, in particular organophosphorus (OP) and carbamate insecticides [14]. All patients were seen regularly at least every 4 h. Significant events (intubation, seizures, death) were recorded prospectively. Patients were also seen on a study ward round twice each day and their condition over the previous 12 h recorded. Patients remained under the care of the hospital consultant physicians. Decisions about medical treatment, intubation, and transfer of patients to intensive care were made solely by the hospital staff on the basis of the patient's clinical condition, and independently of the research team.

### Statistics

Case fatality was defined as the proportion of deaths over admissions for any given pesticide. Odds ratios (ORs) and the 95% confidence intervals (CIs) of the proportions were calculated using the binomial exact method [15]. The binomial exact method will generate a one-sided CI (97.5% CI) when the proportion of deaths over admissions for a given pesticide is zero.

Logistic regression was used to calculate unadjusted and adjusted (for age and gender) ORs for death relative to the pesticide most commonly ingested (chlorpyrifos).

The potential effect of recently implemented pesticide bans was examined assuming proportional redistribution based upon existing distribution of poisoning across the class after excluding patients who had taken unidentified pesticides. Sensitivity analyses were undertaken by assuming complete redistribution to the next most toxic substitute and to the least toxic substitute.

## Results

During the study period, 9,302 patients were admitted following deliberate ingestion of a single pesticide. The pesticide ingested could be identified by history in 7,461 patients; a further 1,841 had ingested an unknown pesticide. Within the unknown group, 497 were considered to have taken a cholinesterase inhibitor (either an OP or carbamate insecticide) on the basis of a typical anticholinesterase clinical syndrome, significant atropine requirement, and/or measured cholinesterase inhibition. A total of 808 patients, whose ingested pesticide was identified by history, had admission samples assayed for other nested studies; history correctly identified the ingested poison in 94.7% of these patients, the other 5.3% were not necessarily misclassifications (more likely to be simply below the level of assay detection). In a subset of the cohort we have also previously shown that analytical assay confirmed history in 92% of symptomatic patients with organophosphate poisoning [16].

The patients were predominately male (67%) and young; the median age was 28 y (interquartile range [IQR] 21–40). The median time to admission to the study hospitals following ingestion was 4 h (IQR 3–7).

The case fatality for the most commonly ingested pesticides is presented in Table 2. The overall mortality for pesticide self-poisoning (including patients who had ingested an unidentified pesticide) was 10.1% (CI 9.5–10.8). From 1 June 2006, 2,608 patients were admitted with a single pesticide ingestion; mortality was 8.9% (61/678) for direct study hospital admissions compared with 7.8% in patients transferred from primary rural hospitals. As 12% of all primary hospital deaths occur in the primary hospitals, there was no clear difference in overall mortality for patients who first presented to a primary rural hospital compared with those presenting to a referral study hospital. Anticholinesterase insecticides and the herbicides paraquat, MCPA, propanil, and glyphosate accounted for 94% of admissions and 98% of deaths. For those ingesting an identified pesticide, just three compounds with relatively high case fatality (paraquat, dimethoate, fenthion) were responsible for 17.6% of total admissions but 47% of the total deaths.

**Table 2:** Case fatality of any pesticide with more than ten admissions or any death.

Pesticide	Deaths	*n* Patients	Percent Case Fatality	95% CI Binomial Exact	Rat Oral LD50 (mg/kg)	WHO Tox Class
**OPs**						
Chlorpyrifos	104	1,376	7.6	6.2–9.1	135	II
Diazinon	4	84	4.8	1.3–11.7	1,000	II
Dimethoate	172	833	20.6	17.9–23.6	c.150	II
Fenthion	35	237	14.8	10.5–19.9	215	II
Malathion	4	209	1.9	0.5–4.8	2,100	III
Methamidophos	1	8	12.5	0.3–52.7	30	Ib
Oxydemeton-methyl	1	8	12.5	0.3–52.7	65	Ib
Phenthoate	11	168	6.5	3.3–11.4	c.400	II
Pirimiphos-methyl	0	12	0.0	0.0–26.5^a^	2,018	III
Profenofos	16	146	11.0	6.4–17.2	358	II
Prothiofos	1	13	7.7	0.2–36.0	925	II
Quinalphos	15	124	12.1	6.9–19.2	62	II
Other OPs^b^	0	38	0.0	0.0–9.3^a^	—	—
**Summary**	**364**	**3,256**	**11.2**	**10.1–12.3**	—	—
**Carbamates**						
Carbaryl	1	18	5.6	0.1–27.3	c.300	II
Carbofuran	5	479	1.0	0.3–2.4	8	Ib
Carbosulfan	37	345	10.7	7.7–14.5	250	II
Fenobucarb	6	104	5.8	2.1–12.1	620	II
Methomyl	1	7	14.3	0.4–57.9	17	Ib
Propoxur	0	16	0.0	0.0–20.6^a^	95	II
Other carbamates^c^	0	7	0.0	0.0–41.0^a^	—	—
**Summary**	**50**	**976**	**5.1**	**3.8–6.7**	—	—
**Pyrethroids**						
Deltamethrin	0	11	0.0	0.0–28.5^a^	c.135	II
Esfenvalerate	1	12	8.3	0.2–38.5	87	II
Etofenprox	1	121	0.8	0.0–4.5	>10,000	IV
Permethrin	0	13	0.0	0.0–24.7^a^	c.500	II
Other pyrethroids^d^	0	46	0.0	0.0–7.7^a^	—	—
**Summary**	**2**	**203**	**1.0**	**0.1–3.5**	—	—
**Organochlorines**						
Endosulfan	2	9	22.2	2.8–60.0	80	II
Lindane	0	3	0.0	0.0–70.8^a^	88	II
**Summary**	**2**	**12**	**16.7**	**2.1–48.4**	—	—
**Other noncholinesterase**						
Abamectin	2	18	11.1	1.4–34.7	650	IV
Acetamiprid	0	11	0.0	0.0–28.5^a^	146	NC
Chlorfluazuron	1	45	2.2	0.1–11.8	8,500	IV
Fipronil	0	26	0.0	0.0–13.2^a^	92	II
Imidacloprid	0	70	0.0	0.0–5.1^a^	450	II
Other insecticides^e^	0	12	0.0	0.0–26.5^a^	—	—
**Summary**	**3**	**182**	**1.6**	**0.3–4.7**	—	—
**Herbicides**						
Alachlor	1	9	11.1	0.3–48.2	930	III
Bispyribac-sodium	3	103	2.9	0.6–8.3	2,635	IV
Fenoxaprop-p-ethyl	0	74	0.0	0.0–4.9^a^	2,357	U
Glyphosate	21	887	2.4	1.5–3.6	4,230	IV
MCPA	33	681	4.8	3.4–6.7	700	III
Oxyfluorfen	0	15	0.0	0.0–21.8^a^	>5,000	II
Paraquat	243	569	42.7	38.6–46.9	150	II
Pretilachlor	0	11	0.0	0.0–28.5^a^	6,100	U
Propanil	45	412	10.9	8.1–14.3	c.1,400	III
Other herbicides^f^	0	22	0.0	0.0– 15.4^a^	—	—
**Summary**	**346**	**2,783**	**12.4**	**11.2–13.7**	—	—
**Fungicides**						
Edifenphos	2	17	11.8	1.5–36.4	150	Ib
Propamocarb	1	1	100	2.5–100	2,000–2,900	IV
Other fungicides^g^	0	31	0.0	0.0–11.2	—	—
**Summary**	**3**	**49**	**6.1**	**1.3–16.9**	—	—
**Unknown pesticides**	173	1,841	9.4	8.1–10.8	—	—

Detail of pesticides included in “Other” groupings, the number of cases in parenthesis following each pesticide.

a Denotes one sided 97.5% CI.

b Acephate (10), coumaphos (4), fenitrothion (2), formothion (1), monochrotophos (10), phoxim (10), terbufos (1).

c Thiodicarb (6), methylcarb (1).

d Cyfluthrin (5), cypermethrin (7), d-trans allethrin (4), fenvalerate (4), flumethrin (6), unspecified pyrethroid (20).

e Azadirachtin (5), chromafenozide (1), flufenoxuron (1), tebufenozide (3), thiacloprid (1), novaluron (1).

f Dinitroaniline (1), ethoxysulfuran (1), methyl 3,4-dichlorocarbanilate (1), dinitroaniline (1), oxadiazon (1), pendimethalin (9), propachlor (1), quinclorac (7).

g Binapacryl (1), carbendazim (2), chlorothalonil (4), dithiocarbamate (7), ethoxysulfan (1), hexaconazole (4), isoprothioline (1), mancozeb (5), propiconazole (1), propineb (1), tebuconazole (2), thiophanate (1), triazole (1).

c., cutaneous; NC Not classified.

**Figure 1:**
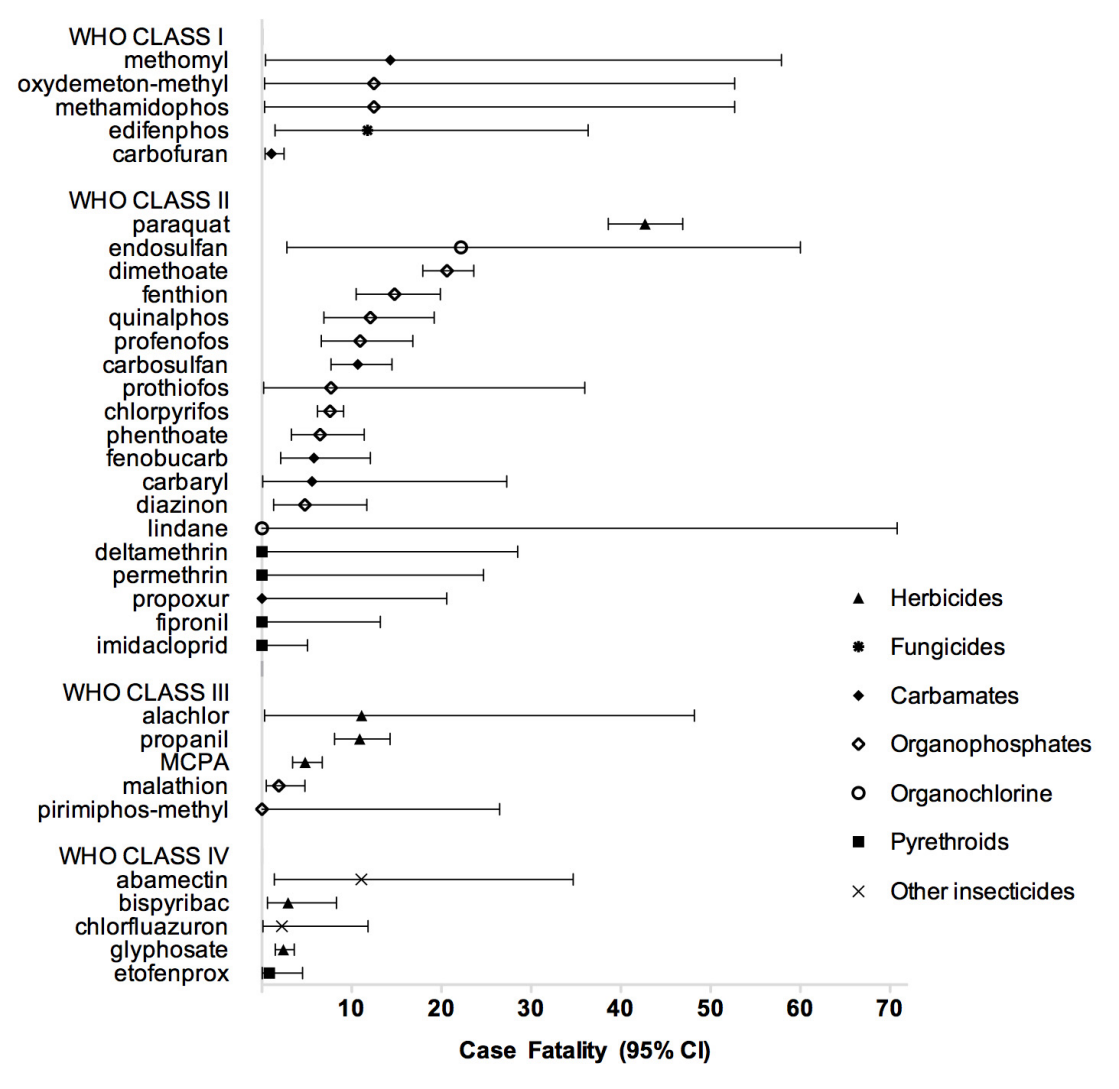
Plot of case fatality for different pesticides grouped by WHO class.

There was a very wide range of lethal toxicity for compounds within the same pesticide classes (e.g., OP insecticides) or with the same agricultural indication (e.g., nonselective herbicides). For example the risk ratio for death after ingestion of the OP insecticides dimethoate versus chlorpyrifos was 2.7 (CI 2.2–3.4) and for the herbicides paraquat versus glyphosate was 18.1 (CI 11.7–27.6). These risk ratios confirm significant differences in pesticides that are in the same WHO class for risk (Figure 1; Table 2) and have comparable agricultural efficacy. There was marked variation in case fatality for OPs with similar LD_50_s, emphasizing the limited usefulness of animal toxicity data to drive human regulatory decisions (Table 2).

Increasing age and male sex was significantly associated with a fatal outcome. However, adjustment for these factors did not result in any major changes in the relative toxicity assessment. For example, the odds of dying from paraquat ingestion was 9.1 (95% CI 7.0–11.8) times that of chlorpyrifos (reference group). When adjusted for age and gender the relative risk of dying from paraquat was moderately greater (OR 15.4; 95% CI 11.6–20.4). Similar moderate increases in ORs were seen for dimethoate and fenthion after adjustment (Table 3).

**Table 3:** Logistic regression of odds of a fatal outcome for each pesticide before and after adjustment for gender and age.

Factor	Unadjusted *n* = 8,883^a^		Adjusted *n* = 8,883	
	Odd Ratio	95% CI	Odd Ratio	95% CI
**Females**	—	—	1.00	—
**Males**	—	—	1.49	1.24–1.80
**<25 y**	—	—	1.00	—
**25–34 y**	—	—	1.48	1.18–1.85
**35–44 y**	—	—	2.39	1.90–3.02
**45–64 y**	—	—	5.86	4.69–7.33
**>64 y**	—	—	19.10	13.45–27.12
**Chlorpyrifos**	1.00	—	1.00	—
**Abamectin**	1.53	0.35–6.74	1.74	0.38–7.99
**Alachlor**	1.53	0.19–12.34	3.00	0.34–26.85
**Bispyribac-sodium**	0.37	0.11–1.18	0.41	0.12–1.35
**Carbaryl**	0.72	0.09–5.46	0.78	0.09–6.64
**Carbofuran**	0.13	0.05–0.32	0.14	0.05–0.34
**Carbosulfan**	1.47	0.99–2.18	1.93	1.28–2.92
**Chlorfluazuron**	0.28	0.04–2.04	0.23	0.03–1.74
**Diazinon**	0.61	0.22–1.70	0.58	0.20–1.69
**Dimethoate**	3.18	2.45–4.13	3.97	3.01–5.23
**Edifenphos**	1.63	0.37–7.23	1.62	0.32–8.24
**Endosulfan**	3.49	0.72–17.04	2.39	0.43–13.25
**Esfenvalerate**	1.11	0.14–8.70	1.65	0.20–13.48
**Fenobucarb**	0.75	0.32–1.75	1.20	0.50–2.84
**Fenthion**	2.12	1.41–3.20	2.91	1.89–4.49
**Glyphosate**	0.30	0.18–0.48	0.42	0.26–0.69
**Malathion**	0.24	0.09–0.65	0.32	0.11–0.89
**MCPA**	0.62	0.42–0.93	0.75	0.49–1.13
**Methamidophos**	1.75	0.21–14.34	1.23	0.14–10.46
**Methomyl**	2.04	0.24–17.09	2.71	0.32–23.15
**Paraquat**	9.12	7.03–11.82	15.42	11.63–20.44
**Phenthoate**	0.86	0.45–1.63	0.92	0.47–1.79
**Profenofos**	1.51	0.86–2.63	1.37	0.76–2.46
**Propanil**	1.50	1.04–2.17	2.06	1.40–3.04
**Prothiofos**	1.02	0.13–7.92	0.88	0.11–7.06
**Quinalphos**	1.68	0.95–2.99	2.18	1.19–3.99
**Unknown pesticides**	1.27	0.98–1.64	1.40	1.08–1.83
**Oxydemeton methyl**	1.75	0.21–14.34	2.42	0.28–21.20
**Etofenprox**	0.10	0.01–0.74	0.08	0.01–0.58

Note of caution: Large OR and wide 95% CI due to small number of deaths in subgroup.

a *n* = 419 omitted from model since no deaths occurred for some categories of pesticide.

The potential effect on mortality of banning the three most lethal pesticides and assuming best and worst case substitutions suggests a reduction in overall pesticide case fatality of between 33% and 65%. The actual fall in case fatality seen after withdrawal of these pesticides will therefore depend substantially on which pesticides replace them in the market (Table 4).

**Table 4:** Modeling the potential effect of dimethoate, fenthion, and paraquat bans in an equal size cohort (7,461 patients with known pesticide^a^ ingestion).

Type of Model	Deaths	Case Fatality	95% CI
No bans (history repeats)	769	10.3%	9.6–11.0%
Proportionate redistribution to other OPs or herbicides^a^	423	5.7%	5.2–6.2%
Least toxic class substitute^b^	351	4.7%	4.2–5.2%
Most toxic class substitute^c^	516	6.9%	6.4–7.5%

a Excludes unknown pesticides.

b Substitution of glyphosate for paraquat as it is the only nonspecific herbicide. Substitution of dimethoate and fenthion with the “Other pesticides” group (using the pooled case fatality).

c Substitution of propanil for paraquat and oxydemeton-methyl for dimethoate and fenthion. Propanil would be an unlikely substitute as it is a selective herbicide.

## Discussion

This study demonstrates major differences in human case fatality between pesticides that are used for similar agricultural indications. These are the first systematically collected prospective human data, to our knowledge, that allow estimates of relative toxicity for pesticides. The data are much more directly relevant to human risk assessment than the existing animal data from which the WHO/EPA classifications of toxicity used in regulation are derived. Moreover, it provides evidence of very large differences in acute human toxicity within these widely used classifications. These data provide a basis for refining further public health, regulatory, and clinical responses to the problem of acute pesticide poisoning.

### Public Health Implications

In regions where pesticide poisoning is a public health problem, pesticide use must be considered in the context of a societal need that balances agricultural benefits with the costs related to human toxicity. Ideally, this requires human toxicity data to be incorporated into regulatory decision making.

This study supports previous observations of the mismatch of human toxicity with animal LD_50_s for OP insecticides [16],[17]. The current WHO/EPA classification is linked to a number of regulatory mechanisms designed to minimize human risk from pesticides on the basis of occupational or unintentional exposure to pesticides [18]. However, the most important current public health problem of pesticides is deliberate rather than unintentional exposure [19]. There is also an overlap between the two in terms of plausible exposure, and therefore acute self-poisoning data also provide substantial information for risk assessment in unintentional exposure.

The criteria for the classifications have provision for the inclusion of other data to supplement the existing standard means of determining classification based on animal data: “Provision is made for the classification of a particular compound to be adjusted if, for any reason, the acute hazard to man differs from that indicated by LD_50_ assessments alone” [18]. The data reported here provide a strong argument for utilizing acute human toxicity data in the existing classification scheme that would be a better guide for national regulatory and promotional policy.

The societal and policy question is what threshold should be adopted to move compounds from a class II (moderately hazardous) status to class 1a (extremely hazardous) or class 1b (highly hazardous) status at which point these compounds would fall under the scrutiny of international regulatory guides and agreements. We have previously suggested that a human case fatality of less than 10% using treatments that are widely available and inexpensive could be the threshold for classification of a pesticide formulation into class II [8].

Alternatively, rather than arbitrarily setting a threshold, benchmarking could utilize data on a range of class I agents. In a retrospective Indian study, class I pesticides had case fatality ranging from 17% to 60%, and no class II pesticides were in this range [17]. Thus one might set the threshold at a human case fatality of less than 15% to remain in class II. From a public health perspective it seems irrational for pesticide formulations with estimated case fatalities of 21% (dimethoate EC40), 22% (endosulfan EC35), and 47% (paraquat 20%) to be in the same (moderately hazardous) WHO class as many pesticides with a 20-fold lower human case fatality (see Table 2, case fatality for other class II pesticides cluster in the range 0% to 12.5%).

The public health impact of such a change in classification is supported by past experience of pesticide restriction. In Sri Lanka, the withdrawal of all class I pesticides in the years leading up to 1995 and withdrawal of endosulfan in 1998 coincided with a halving of the number of suicides and even greater reduction in fatal poisonings [7],[20]. These pesticide restrictions were introduced without any adverse effects on agricultural costs or output [21]. In January 2008, the data presented within this paper combined with other studies [22] underpinned a Sri Lankan government policy decision to withdraw paraquat, dimethoate, and fenthion from the Sri Lankan market. Our analysis (Table 3) suggests the decision to withdraw these three pesticides is likely to lead to a further 33% to 65% reduction in deaths from acute poisoning. On the basis of Sri Lankan 2005 figures for deaths from poisoning, this action should translate to a reduction in suicide deaths by about 1,000 lives per year [7]. Similar regulatory activities across Asia would likely reduce the suicide rate in the region by more than 30% contributing to a substantial fall in the global number of suicides.

### Clinical Implications

The large variation in mortality within OP and carbamate pesticides suggests variation in toxicokinetics and/or dynamics for compounds that are often regarded as requiring the same medical management [16]. Clinical implications of significant variability within a toxicological class include the need for a more complex risk assessment for individual patients and perhaps specific treatment protocols for individual agents [16]. Previous analysis of the relative toxicity of just three OP insecticides (chlorpyrifos, dimethoate, and fenthion) demonstrated large difference in the time course of toxicity, kinetics and dynamics of the pesticide, and mode of death [16]. Better understanding of factors that are associated with higher mortality will allow clinicians to triage and manage patients more effectively. While this analysis has focused simply upon differences in case fatality, further studies are required to provide more detail on the clinical course of high risk poisonings.

### Effects of Resource Limitations in Rural Asian Hospitals

The limited treatment resources in Sri Lankan primary referral hospitals might have increased case fatality [23]. However, such limitations are the norm throughout the developing world. For example, propanil's case fatality of 11% is unexpectedly high for an agent whose toxicity is largely due to methaemoglobinaemia and haemolysis, complications that should be relatively easily treated [24]. However, the difficulty in monitoring treatment response without cooximeters and problems with access to and dose adjustment of the antidote have all been suggested as major contributory causes to the high case fatality [24]. Previous studies have also documented high complication rates from gastrointestinal decontamination in rural hospitals that may increase mortality [25]. Other issues identified in primary hospitals that could impact on care include lack of medication, equipment, and staffing [23]. Resource restrictions are less likely to have an effect on case-fatality estimates for highly toxic pesticides such as paraquat that have no proven effective treatment [22],[26].

These two study hospitals received additional resources in the form of medically qualified research assistants and additional monitoring equipment. These and standardized treatment protocols would be expected to have contributed to a generally lower case fatality than other rural hospitals [27],[28], and thus we believe the estimates of actual lethality and the potential impact of further regulation are both conservative. In addition, for 3 y of this study, dimethoate and fenthion were banned in approximately 25% of the province, contributing to an underestimate of the effects of more widespread pesticide restriction.

### Effect of Pesticide Formulation

For some pesticides in particular, mortality may not be just related to the intrinsic toxicity and whether effective antidotes have been developed. Formulation differences can be important. The acute toxicity of the class I agent carbofuran is far higher (rat oral LD_50_, 8 mg/kg) than the related carbosulfan (250 mg/kg). However, carbofuran has remained on the market as it is sold as 3% granules and therefore has significantly lower case fatality than carbosulfan that is formulated as a 25% emulsifiable concentrate liquid. The benefits of this formulation change have been attenuated by farmers using the granules “off label” to produce a solution. The OP quinalphos has the highest acute rat toxicity of any OP sold in Sri Lanka; as a result, it is formulated at a lower concentration (25%) than most other OPs (40%–50%) to bring its predicted toxicity down. Unfortunately, changes in formulation for paraquat have not led to clinically significant reductions in case fatality [22]. Formulation changes have not yet been attempted for other pesticides.

### Comparison with Other Research Studies

There are no other comparable prospective studies of pesticide mortality but previous retrospective studies support our key conclusion that pesticides used for the same agricultural need have very important differences in acute human toxicity.

A retrospective study in South India of 8,040 pesticide ingestion showed an overall mortality from OP insecticides of 22% [17] compared to 11.1% in this study (Table 2). The majority of deaths were due to the class I OP monocrotophos, which had a case fatality of 35%. Similar data have been reported from a retrospective poison center study in Taiwan [29]. Class I OPs in these studies showed higher mortality than all of the class II or III OPs in our study with the exception of dimethoate. While the overall mortality in our cohort was much lower, the results for the few pesticides that were common to these studies were similar despite the different setting; the case fatality in the Indian study was chlorpyrifos (6%), quinalphos (12%), and endosulfan (28%), and in the Taiwan study was chlorpyrifos (4.4%), dimethoate (16.6%), and fenthion (15.3%).

These studies provide strong support for our contention that these results are representative of typical poisonings in the region. However, since relatively few pesticides overlapped, there is a clear need for more large, prospective cohort studies in the region to inform pesticide regulation across the full range of pesticides in agricultural use.

### Limitations

The most important limitation of our data is that they were collected from just two rural referral hospitals in one country. However, these hospitals are likely to be representative for many rural hospitals in the developing world where resources may be restricted. Although data were collected from within one country, the pattern of agriculture and related pesticide requirements within the study area are similar to much of rural Asia [30].

Since Sri Lanka has already banned some pesticides (notably all WHO class I pesticides [31]), these data do not include some WHO class I pesticides (such as parathion and monocrotophos) that are still manufactured and available in other countries of the region. The limited human data on such agents suggest they are more toxic than most of the pesticides in our study [29]. It is likely that any country developing a policy based upon including pesticides of minimum risk in acute exposure would first attempt to regulate these class 1 agents.

Another potential limitation is that these data on deaths and poisonings were recorded from secondary referral hospitals. Deaths at home, in primary hospitals, and during transfers will affect both the numerator and denominator of the observed case fatality. Surviving patients not transferred will only reduce the denominator. Data collected for a previous study of all injury in the province identified only 3.8% of deaths from pesticides occurring outside the health system (Table S1) [32]. Our data would not include patients who took trivial nontoxic ingestions of agrochemicals and did not subsequently present for medical treatment. If this occurred it should be more common with the least toxic pesticides and thus to reduce differences observed between agents. It thus could not explain the very large differences in relative toxicity we observed.

We examined primary hospital mortality within two subsets of this cohort. In a small study of 260 patients done in 2002 from within this cohort, 80% of deaths occurred after transfer [12]. Of 471 patients presenting with pesticide poisoning to peripheral hospitals in a 6-mo period in 2005, just three patients died in the peripheral hospital compared to 32 patients dying after transfer. 77% of organophosphate, 84% of carbamate, and 90% of paraquat poisoned patients were transferred to the study hospitals (Table S2) [13].

We prospectively examined all poisonings presenting to all primary hospitals (*n* = 30) and referral hospitals between September 2008 and December 2009 as part of an RCT of primary hospital educational intervention (ISRCTN73983810). 1,158 people with pesticide poisoning presented to the primary rural hospitals; 77% of these patients were transferred to the referral hospitals. A total of 72 deaths occurred within this group, nine deaths were in the primary hospital, and 63 deaths in referral hospital. (Table S3). Median time to presentation in this study was 55 min (IQR 30–110).

As most deaths occur following referral it is unlikely that patients kept in primary hospitals and not referred would substantially change the overall or relative case fatality. Any bias from nonreferral of less severely poisoned patients would lead to overestimation of toxicity for the least toxic pesticides. None of the data presented (Tables S1, S2, S3) suggests any systematic difference in pesticides causing death in the referral hospital compared with prereferral (primary hospitals and home). Thus this potential referral bias could not explain the very large differences in case fatality between pesticides that we found.

### Future research

It is possible that other pesticides with apparently low case fatality based on small numbers may later emerge as important agents in poisoning as their agricultural use in rural Asia increases [20]. This possibility mandates a need for ongoing “toxico-vigilance” studies to follow any change in regulation. It is possible that examination of other important but nonfatal clinical outcomes may also help provide better estimates of the likely human fatal toxicity for these agents. While it is intuitive that a poisoning that never causes symptoms is less likely to be lethal than one that has caused major complications, but no deaths, the methodology for quantifying this hypothesis has not been developed.

### Conclusion

There is marked variation in human lethality from acute poisoning within the existing pesticide classifications. Accounting for this in promotional, regulatory, and clinical decision making is likely to have a major impact upon overall mortality from suicide with pesticides and global suicide numbers.

## Supporting Information

Table S1 Place of death in Anuradhapura district determined by triangulation of data from hospital, coronial, and police records for pesticide poisoning.
(0.04 MB DOC)

Table S2 Primary hospital data from Anuradhapura District for 6 mo (2005) detailing the patients presenting to primary rural hospitals.
(0.03 MB DOC)

Table S3 Outcome for patients admitted to primary hospitals following pesticide poisoning in Anuradhapura district between September 2008 and December 2009. Deaths occurring after primary hospital transfer include six deaths during transport to the referral hospital; the remainder of deaths occurred in the referral hospital.
(0.04 MB DOC)

## Abbreviations

CI: confidence interval
EPA: Environmental Protection Agency
FAO: Food and Agriculture Organization
IQR: interquartile range
OP: organophosphorus
OR: odds ratio
WHO: World Health Organization
