# Supplementary material for: Acute Human Lethal Toxicity of Agricultural Pesticides: A Prospective Cohort Study
Source: PLoS Med. 2010 Oct 26;7(10):e1000357. doi: 10.1371/journal.pmed.1000357 (PMC2964340; doi:10.1371/journal.pmed.1000357)
Supplement: Table S1 — Place of death in Anuradhapura district determined by triangulation of data from hospital, coronial, and police records for pesticide poisoning. (0.04 MB DOC) [file pmed.1000357.s001.doc]

|  | Place of Death | | | | | |  |
| --- | --- | --- | --- | --- | --- | --- | --- |
| Type of Pesticide | Home | Primary Hospital | Out of Province | During transfer to referral hospital | Referral Hospital | Unknown | Total Deaths |
| Carbamate |  | 3 |  | 3 | 1 | 1 | 8 |
| Organophosphate | 2 | 2 |  |  | 19 |  | 23 |
| Other Herbicide |  |  |  |  | 5 |  | 5 |
| Other Insecticide |  |  |  |  | 5 |  | 5 |
| Paraquat | 1 |  |  | 1 | 27 | 2 | 31 |
| Unknown pesticide |  | 1 | 1 |  | 2 | 1 | 5 |
|  |  |  |  |  |  |  |  |
| Total | 3 | 6 | 1 | 4 | 59 | 4 | 77 |

Supplementary Table 1

Place of death in Anuradahpura district determined by triangulation of data from hospital, coronial and police records for pesticide poisoning. From data set and methods described in Eddleston [1]

1. Eddleston M, Udayakumara N, Adhikari S, de Silva D, Sheriff MH, et al. (2007) The importance of poisoning vs. road traffic injuries as a cause of death in rural Sri Lanka. PLoS One 2: e599.
