# Supplementary material for: Acute Human Lethal Toxicity of Agricultural Pesticides: A Prospective Cohort Study
Source: PLoS Med. 2010 Oct 26;7(10):e1000357. doi: 10.1371/journal.pmed.1000357 (PMC2964340; doi:10.1371/journal.pmed.1000357)
Supplement: Table S2 — Primary hospital data from Anuradhapura District for 6 mo (2005) detailing the patients presenting to primary rural hospitals. (0.03 MB DOC) [file pmed.1000357.s002.doc]

| Type of Pesticide | Number of Patients admitted | Transferred to referral hospital | %  transfers | Deaths in primary hospital | Deaths in referral hospitals |
| --- | --- | --- | --- | --- | --- |
| Carbamate | 50 | 42 | 84% | 0 | 2 |
| Organophosphate | 157 | 121 | 77% | 2 | 15 |
| Glyphosate ^1^ | 46 | 31 | 67% | 0 | 1 |
| MCPA^1^ | 32 | 23 | 72% | 0 | 1 |
| Other Pesticide^2^ | 165 | 114 | 69% | 0 | 8 |
| Paraquat^1^ | 21 | 19 | 90% | 1 | 5 |
| Total | 471 | 350 | 74% | 3 | 32 |

Supplementary Table 2

Primary hospital data from Anuradahpura District for 6 months 2005 from Senarathna [2] detailing the patients presenting to primary rural hospitals.

^1^ Herbicides

^2^ Includes unknown pesticides and pesticides otherwise not classified

2. Senarathna L (2006) How the level of resources and hospital staff attitude in primary care hospitals in rural Sri Lanka affect poisoning patient outcome. University of Newcastle. http://www.sactrc.org/thesis.html
