# Supplementary material for: Acute Human Lethal Toxicity of Agricultural Pesticides: A Prospective Cohort Study
Source: PLoS Med. 2010 Oct 26;7(10):e1000357. doi: 10.1371/journal.pmed.1000357 (PMC2964340; doi:10.1371/journal.pmed.1000357)
Supplement: Table S3 — Outcome for patients admitted to primary hospitals following pesticide poisoning in Anuradhapura district between September 2008 and December 2009. Deaths occurring after primary hospital transfer include six deaths during transport to the referral hospital; the remainder of deaths occurred in the referral hospital. (0.04 MB DOC) [file pmed.1000357.s003.doc]

| Type of Pesticide | Death in Primary Hospital | Discharged from primary hospital | Left against medical advice | Missing patient chart | Transferred to referral hospital | Death after primary hospital transfer | Total |
| --- | --- | --- | --- | --- | --- | --- | --- |
| Carbamate | 1 | 27 | 4 |  | 94 | 3 | 126 |
| Organophosphate | 4 | 53 | 9 | 1 | 313 | 22 | 380 |
| Other herbicide | 2 | 83 | 9 |  | 278 | 24 | 372 |
| Other insecticide |  | 15 | 4 | 1 | 63 | 4 | 83 |
| Paraquat |  | 6 | 1 |  | 38 | 5 | 45 |
| Unknown Pesticides | 2 | 35 | 4 | 1 | 110 | 5 | 152 |
|  |  |  |  |  |  |  |  |
| Total | 9 | 219 | 31 | 3 | 896 | 63 | 1158 |

Supplementary Table 3

Outcome for patients admitted to primary hospitals following pesticide poisoning in Anuradapura district between September 2008 and December 2009. Deaths occurring after primary hospital transfer include 6 deaths during transport to the referral hospital, the remainder of deaths occurred in the referral hospital
